# Validation of the irritation scale on a representative German sample: new normative data

**DOI:** 10.1038/s41598-023-41829-4

**Published:** 2023-09-16

**Authors:** Maria S. Gralla, Harald Guendel, Andreas Mueller, Elmar Braehler, Winfried Häuser, Johannes Kruse, Beate Muschalla, Thomas Rigotti, Bernhard Strauss, Elisabeth M. Balint

**Affiliations:** 1 Department of Psychosomatic Medicine and Psychotherapy, Clinical Center, Ulm University, Albert-​Einstein-Allee 23, 89081 Ulm, Germany; 2 Institute of Psychology, Work- and Organizational Psychology, University of Duisburg-Essen, Duisburg, Germany; 3 Department of Psychosomatic Medicine and Psychotherapy, University Medical Center of the Johannes Gutenberg University of Mainz, Mainz, Germany; 4 Research and Treatment Center Adiposity Diseases, Behavioral Medicine Unit, Department of Psychosomatic Medicine and Psychotherapy, Leipzig University Medical Center, Leipzig, Germany; 5 Department Psychosomatic Medicine and Psychotherapy, Technische Universitaet Muenchen, Muenchen, Germany; 6 Department of Psychosomatic Medicine and Psychotherapy, University of Giessen, Giessen, Germany; 7 Department of Psychosomatic Medicine and Psychotherapy, University of Marburg, Marburg, Germany; 8 Institute of Psychology, Psychotherapy and Diagnostics, Technische Universitaet Braunschweig, Braunschweig, Germany; 9 Work, Organizational and Business Psychology, Johannes Gutenberg University, Mainz, Germany; 10 Leibniz Institute for Resilience Research, Mainz, Germany; 11 Institute of Psychosocial Medicine, Psychotherapy and Psycho-Oncology (IPMPP), Jena, Germany; 12 Center for Burnout and Stress-Related Disorders, Privatklinik Meiringen, Meiringen, Switzerland

**Keywords:** Psychology, Occupational health

## Abstract

The irritation scale is a widely used and reliable self-report scale for measuring cognitive and emotional strain related to the work environment. It extends existing measures by providing a sensitive assessment for pre-clinical stress at work. Existing normative data are based on convenience samples and are therefore not representative. This study provides new normative data for the irritation scale based on a representative German sample (*N* = 1480). The new normative data indicate that the overall level of irritation in the German workforce is significantly lower compared to previously published data. Convergent and discriminant validity is confirmed by correlations with depression and anxiety (Patient Health Questionnaire-4 for Depression and Anxiety), somatic symptom scales (Bodily Distress Syndrome 25 checklist, Somatic Symptom Scale-8, Giessen Subjective Complaints List-8, comorbidity), psychological functioning (Mini-ICF rating for activity and participation disorders in mental illness), work-related stressors (overcommitment and bullying) and individual resources (self-efficacy). The results confirm the utility of the irritation scale and provide new benchmarks that avoid an underestimation of the levels of irritation in future studies.

## Introduction

Instruments that assess work-related psychological strain are important for identifying risks for health impairments and unfavorable work design. If these are detected at an early stage, preventive action can be taken. In the workplace context, preventing disease is more cost-effective than treating it^1^. In addition, it has a positive impact on the quality of life of employees^2^. The concept of irritation is an established indicator for the assessment of psychosocial strain at work^3^. It was first introduced by Mohr and is based on the assumption that mental impairment results from a perceived goal discrepancy, which is expressed by both increased mental preoccupation with work-related issues (cognitive irritation, CI) and irritability (emotional irritation, EI)^4^. Both aspects are captured by the irritation scale (IS)^5^. The IS is a frequently used instrument that is able to identify psychological strain at work at an early stage^6,7^. Based on the developmental model of impairment of psychological well-being^8^, it is considered a mediator between (social) stressors and depressive symptoms^9^. The scale is recommended for the evaluation of interventions^10–12^ and for the early detection of psychosomatic complaints^13^, among others. The eight items of the irritation scale have been translated and validated in several different languages^3^. Mohr, Müller et al. provided normative data of the irritation scale by analyzing data from 20 different data sets (total *N* = 4030 individuals)^6^. The data sets were convenience samples with partly high strain occupational groups (e.g. police officers and firefighters). The aim of this paper is to present current norms, review the factorial structure of the scale and present both new and confirmatory evidence regarding construct validity of the irritation scale based on a representative German sample. Since the first normative data of the IS scale presented by Mohr et al. was not based on a representative sample, which could have distorted the correlations with validity measures (e.g., Warr^14^), we examine the construct validity on the base of data representative for working adults in Germany. For this purpose, correlations with clinical indicators (depressive and anxious symptoms, psychosomatic complaints, psychological functioning), with work stressors (overcommitment, bullying), and with individual resources (self-efficacy) were considered.

### Specific hypotheses regarding construct validity

From the perspective of theoretical models of goal discrepancy and mental health^15,16^, irritation is regarded as a non-clinical precursor of clinical mental impairments. It can therefore be assumed that irritation has a positive correlation with clinical symptoms such as depression, anxiety, somatic complaints and reduced general mental functioning.

To the author's knowledge, only subclinical depression measures have been used to date to evaluate the association of IS with depressive symptoms^9,17^. The Patient Health Questionnaire for Depression and Anxiety (PHQ)-4^18^ has been established as an ultra-brief, excellent screening tool of depression and anxiety. In the presented data, its relationship to irritation will be examined. According to the results of Dormann et al., a medium to high positive correlation is expected^9^.

Possible correlations with somatic complaints are also of interest. These are often the trigger for seeking medical help when psychological problems such as depression are present and can be evaluated as an alarm signal for high stress^19^. A medium positive correlation of the IS with various somatic complaints is expected, based on the correlations found so far by Müller et al. with psychosomatic complaints^16^. In this study, we use different instruments to record psychosomatic complaints (Bodily Distress Syndrome 25 checklist, Somatic Symptom Scale, Giessen Subjective Complaints List, comorbidity).

When mental and somatic difficulties occur, it usually affects functioning in everyday life^20^. Thus, if the IS is related to depression, anxiety, and somatic complaints, a relationship to functioning is also expected. The Mini-ICF rating for activity and participation disorders in mental illness (Mini-ICF-APP-S)^21^ captures illness-related ability impairment and covers more areas than the PHQ-4 or the somatic scales. A positive relationship can be assumed.

In addition to the presumed relationships with clinical factors, Irritation is considered a medium-term consequence of work-related stressors^3^. Bullying or mobbing is one of the work stressors associated with the highest risk for mental health, as it systematically undermines social resources^22^. Thus, a positive correlation of IS with bullying, measured with the Mobbing Intensity of Colleagues (MOB-C)^23^, can also be expected. Also, the relationship with work-related overcommitment^24^ will be investigated, which has not been reported in the literature so far. A very high positive correlation is expected, especially with the subscale CI, since the constructs overlap strongly.

Moreover, self-efficacy is thought as a personal resource that acts as a mediator between stress and mental health^25^. Accordingly, high values of IS are expected to be accompanied by low self-efficacy values^16^.

In addition to the presumed direction and strength of the correlations, certain assumptions can be made about the extent to which the subscales EI and CI correlate differently with the respective constructs. From the model of goal discrepancy^15^ it can be concluded that EI shows a closer correlation to clinical psychological symptoms than CI: Ruminations in the sense of CI are seen as direct reactions to a perceived goal discrepancy resulting from work stressors, for example. Here, CI represents the cognitive simulation of possible solution strategies to overcome this discrepancy. If efforts to reach the goal remain in vain, frustration and irritability follow in the sense of EI, which is thus seen as a direct precursor to mental diseases. In this respect, we suspect stronger associations of all clinical constructs with the EI subscale, compared to CI.

## Materials and methods

### Sample characteristics

The present study was based on a representative survey on physical and mental well-being in the German population. Data were collected by USUMA (Independent Service for Surveys, Methods and Analyses, Berlin) between May and July 2019. Written informed consent was obtained from all the study participants. The study was approved by the Institutional Ethics Review Board of the University of Leipzig (No. 145/19-ek) and all experiments were performed in accordance with relevant guidelines and regulations. The sample consisted of a total of 2531 persons. The sample included 1350 females and 1181 males with an average age of 48.44 (*sd* = 17.86). For more details on the sociodemographic characteristics of the study sample see the supplementary table. The survey followed ADM (Arbeitskreis Deutscher Markt-und Sozialforschungsinstitute e.V.) sampling guidelines for generating a representative sample of the German population^26^. Weighting variables provided by USUMA were applied, which correct for the increased selection probability of individuals in small households and the distortions due to a lack of willingness to participate on the part of randomly selected individuals. All analyses were calculated with the weighted values. Participants with missing values of the IS were excluded (*n* = 1020). Since the IS is a work-related instrument, only persons with valid IS-values who worked at least 15 h per week (*n* = 1406) were included in the analysis. Offsetting this with the sampling weights yields a new *N* = 1480 for all further analyses. For calculating the internal consistency of the other scales, the full sample size of *N* = 2531 was used.

### Irritation scale (IS)

The IS contains eight items answered on a scale between 1 (strongly disagree) and 7 (strongly agree). The two primary-order factors emotional irritation (EI) (sum score of items 3, 5, 6, 7, 8; range from 5 to 35) and cognitive irritation (CI) (sum score of items 1, 2, 4; range from 3 to 21) add to the second-order factor ‘general irritation’ (GI), ranging from 7 to 56, with higher values indicating higher irritation (items see Table 1)^5^. The two-factorial structure of the scale was confirmed by Müller et al. using exploratory and confirmatory factor analysis^16^ using latent variable modeling techniques with structural equation modeling as recommended by Rodriguez and Reise^27^. Müller et al. showed that a two-factorial model of the Irritation Scale showed a better fit to the data than a competing one-factorial model. The two-factorial structure was confirmed in a further study by Mohr et al.^3^ for nine language adaptions of the same scale.

**Table 1:** Means, standard deviations, skewness, kurtosis and corrected item-total correlation (N = 1480).

Item	*M*	*SD*	Skewness	Kurtosis	Corrected item-total correlation
(1) I have difficulty relaxing after work. (CI)	2.49	1.55	0.91	− 0.10	0.71
(2) Even at home I often think of my problems at work. (CI)	2.50	1.54	0.86	− 0.16	0.76
(3) I get grumpy when others approach me. (EI)	2.13	1.33	1.18	0.81	0.75
(4) Even on my vacations I think about my problems at work. (CI)	1.97	1.39	1.43	1.17	0.74
(5) From time to time I feel like a bundle of nerves (EI)	1.80	1.22	1.57	1.74	0.74
(6) I anger quickly (EI)	2.20	1.35	1.12	0.65	0.72
(7) I get irritated easily, although I don’t want this to happen. (EI)	2.21	1.37	1.15	0.75	0.77
(8) When I come home tired after work, I feel rather irritable. (EI)	2.02	1.28	1.28	0.95	0.76

*CI* Cognitive irritation, *EI* emotional irritation. Scale between (1) = strongly disagree and (7) = strongly agree.

We performed a confirmatory factor analysis (CFA) to analyze the dimensional structure of the theoretical model using the statistical package AMOS 26^28^. Since AMOS cannot consider case weights, we calculated the CFA with unweighted values (*N* = 1406). We analyzed three different models, using the Asymptotically Distribution-Free estimation procedure, due to the non-normally distributed data: A single-factor model where all items load on one general factor (GI), a two oblique factors solution where the respectively hypothesized items load on the two correlated latent factors CI and EI and a bifactorial model, where all items load on GI as well as the respectively hypothesized items on the two correlated latent factors CI and EI. Model fit was estimated with accepted fit indices Chi-square, CFI and RMSEA using the criteria suggested by Hu and Bentler^29^ who suggested values > .95 for CFI, and < 0.06 for RMSEA indicating a good fit. To compare the models, we calculated a Chi-square difference test using ∆Chi-square and ∆*df,* and looked at ∆CFI and ∆RMSEA. For ∆CFI a meaningful difference is assumed from < − 0.01 and for ∆RMSEA > 0.015^30^.

### Psychometric instruments

#### Patient Health Questionnaire for Depression and Anxiety (PHQ-4)

The Patient Health Questionnaire for Depression and Anxiety (PHQ-4) assesses depressive and anxious symptoms with four items on a scale from 0 (not affected at all) to 3 (affected almost every day) within the last two weeks^18^. Cronbach's alpha for this sample is 0.86.

#### Bodily Distress Syndrome 25 Checklist (BDS 25)

The Bodily Distress Syndrome 25 Checklist (BDS 25) uses 25 items on a scale of 0–4 to diagnose bodily distress syndrome, an overarching diagnosis proposed in 2010 for so-called functional somatic syndromes within the four categories of cardiopulmonary, gastrointestinal, musculoskeletal, and general symptoms^31^. Cronbach's alpha is 0.93.

#### Somatic Symptom Scale-8 (SSS-8)

The Somatic Symptom Scale-8 (SSS-8) is a short form of the Patient Health Questionnaire PHQ-15 and measures impairment by eight different somatic symptoms within the past four weeks on four factors (gastrointestinal, pain, cardiopulmonary, and fatigue) on a scale of 0–4^32^. Cronbach's alpha is 0.86.

#### Giessen Subjective Complaints List (GSC-8)

The Giessen Subjective Complaints List (GSC-8) assesses current complaints on a scale of 0–4 using eight different items that can be assigned to four factors: gastrointestinal, musculoskeletal, fatigue, and cardiovascular complaints^33^. Cronbach's alpha is 0.89.

#### Comorbidity

The Comorbidity scale captures current impairment from 13 different health problems (e.g., hypertension, diabetes, cancer) using a dichotomous response format. It is part of the German version of the Self-Administered Comorbidity Questionnaire (SCQ-D)^34^. Cronbach's alpha of the reported sample is 0.80. For a comparison and discussion of somatic complaint scales see the work by Häuser et al.^35^.

#### Mini-ICF rating for activity and participation disorders in mental illness (Mini-ICF-APP-S)

The Mini-ICF rating for psychological activity and participation disorders in mental illness is used in the self-report version (Mini-ICF-APP-S)^21,36^. The assessment based on the International Classification of Functioning, Disability and Health ICF^37^ captures the subjective assessment of thirteen soft skills on a scale from 0 (‘This is clearly a strength of mine’) to 7 (‘I cannot do this at all’). In this work, the following dimensions are considered: (1) adjust to rules and routines, (2) plan and structure tasks, (3) be flexible, (4) apply knowledge, (5) make decisions, (6) be proactive, (7) be enduring, (8) be assertive, (8) interact with other persons, (10) integrate in groups, (11) build dyadic relationships, (12) self-care, and (13) move around^27^, which are combined into superordinate factors: cognitive and soft-skills (1–7), interactional skills (8–11) and basic skills (12, 13). Cronbach's alpha is 0.92.

#### Mobbing Intensity of Colleagues (MOB-C)

The Mobbing Intensity of Colleagues (MOB-C) scale consists of four items which ask about the subjective experience of workplace bullying on a scale of 1 (strongly disagree) to 4 (strongly agree)^23^. Cronbach's alpha is 0.91.

#### Overcommitment scale (OC)

The Overcommitment scale (OC) is part of the Effort-Reward Imbalance scale (ERI) and measures the tendency to be overcommitted at work with six items on a scale from 1 (strongly disagree) to 4 (strongly agree)^38^. For the reported sample, Cronbach's alpha is 0.70.

#### General Self-efficacy in Easy Language (GSE-EL)

This questionnaire captures General Self-Efficacy in easy language (GSE-EL)^39^. The GSE-EL asks about optimistic competence expectations, i.e., the confidence in mastering difficult situations with the help of one's own competences^39^. Compared to the original scale from Jerusalem and Schwarzer^40^, the wording of the 10 items used here was simplified by Berger and colleagues^39^ and a dichotomous response format was chosen (0 = no; 1 = yes) and item six was rephrased. Cronbach's alpha is 0.84.

### Statistical analyses

Data were analyzed using IBM SPSS Statistics version 27. For the new normative values, the respective percentile rank values were calculated for the raw scale values of the two subscales EI and CI as well as for the GI. Pearson correlations were calculated for testing discriminant and convergent validity. If inferential tests are used, exact *p* values, effect sizes, and 95% confidence intervals (95% CI) are reported. Due to the size of the sample, parametric tests were calculated despite the lack of a normal distribution. Tests were performed two-sided at a significance level of *p < *0.05.

## Results

Table 1 shows means, standard deviations, skewness and kurtosis of the individual IS items. All items show substantially positive skewness. For a positively skewed distribution most data falls to the right of the graph's peak^41^. A Shapiro Wilk test contradicts the assumption of normal distribution of the data. Internal consistency is considered very high with a Cronbach's alpha of 0.92 for the GI, 0.89 for CI and 0.91 for EI. The correlation of the subscales EI and CI is *r = *0.702, *p < *0.001; 95% CI [0.675; 0.727].

### Factor analysis

An exploratory factor analysis confirmed the bifactorial structure of IS in our sample: 71.2% of the variance is explained by the first two factors (61.9% by factor 1 and 9.3% by factor 2). The oblimin-rotated factor matrix results in an assignment of the items to the two factors (items 1, 2 and 4 to factor 1, items 3, 5, 6, 7 and 8 to factor 2). The correlation of the factors is *r = *0.664. To further examine the factor structure, we calculated a CFA testing a single-factor model, a two oblique factor model and a bifactor model. The model fit indices are presented in Table 2. The single factor model did not reach an overall good fit. The two-factor solution appeared to be superior compared to the single-factor model. However, the fit-indices of the two-factor model are contradictory. While the RMSEA indicate reasonable fit, the CFI indicate a rather poor fit, according to conventional cut-off criteria. The two latent factors CI and EI correlate with *r = *0.828, the covariance *s*_CIEI_ is 1.00, *S.E. = *0.058. The two-factor model fits the data significantly better than the single factor model according to the Chi-square difference test (∆χ^2^* = *44.08, ∆*df = *1, *p < *0.001), as well as ∆CFI* = *− 0.11 and ∆RMSEA* = *0.02. The bifactorial model did not converge.

**Table 2:** Indizes of the confirmatory factor analysis of the single-factor and the two-factor model.

Model	χ^2^	*df*	CFI	RMSEA	RMSEA 90% CI
Single factor (GI)	249.22***	20	0.65	0.10	[0.089; 0.109]
Two oblique factors (CI, EI)	205.14***	19	0.76	0.08	[0.073; 0.094]

*Df* degrees of freedom, *CFI* Comparative Fit Index, *RMSEA* Root Mean Square Error of Approximation, *90% CI* 90% confidence interval, *GI* general irritation, *CI* cognitive irritation, *EI* emotional irritation.

***p < 0.001.

The standardized parameter estimates of the single-factor and the two-factor solution are presented in Table 3. Overall, the factor loadings are consistently high.

**Table 3:** Undstandardized and standardized factor loadings of the single-factor and the two-factor model.

Single-factor model		GI							
		IS1	IS2	IS3	IS4	IS5	IS6	IS7	IS8
	Unstandardized λ	1.00	1.06	0.87	0.87	0.78	0.90	0.99	0.91
	*S.E*	–	0.03	0.03	0.03	0.04	0.04	0.04	0.03
	Standardized λ	0.84	0.89	0.82	0.84	0.81	0.86	0.92	0.84

Two-factor model		CI			EI				
		IS1	IS2	IS4	IS3	IS5	IS6	IS7	IS8
	Unstandardized λ	1.00	1.09	0.87	1.00	0.88	1.06	1.16	1.04
	*S.E*	–	0.03	0.03	–	0.04	0.03	0.03	0.04
	Standardized λ	0.86	0.92	0.84	0.83	0.80	0.86	0.92	0.84

*GI* General irritation, *λ* factor loading, *S.E.* standard error, *CI* cognitive irritation, *EI* emotional irritation.

### New norms

Table 4 shows the raw scores with the corresponding percentile rank values for the two subscales and the overall index.

**Table 4:** Raw scores and percentiles for the subscales CI and EI and GI.

Raw scores	CI	EI	GI
3	26.5		
4	36.4		
5	47.5	25.0	
6	57.1	34.4	
7	63.9	41.2	
8	69.4	47.2	17.1
9	75.9	54.9	22.2
10	80.6	62.0	29.1
11	85.3	68.0	33.9
12	88.1	71.5	39.1
13	90.7	75.5	43.2
14	93.5	79.2	48.6
15	95.6	81.5	51.9
16	97.2	83.6	56.1
17	98.5	86.8	60.9
18	99.0	89.1	64.0
19	99.3	90.4	67.1
20	99.7	92.4	70.7
21	100.0	94.9	72.6
22		96.4	75.3
23		97.1	77.1
24		98.0	79.1
25		98.2	81.6
26		99.0	83.6
27		99.3	85.3
28		99.5	87.5
29		99.7	88.6
30		99.8	89.6
31		99.9	91.0
32		100.0	92.1
33		100.0	93.1
34			94.1
35			94.7
36			95.5
37			96.1
38			97.3
39			97.6
40			98.3
41			98.7
42			99.1
43			99.2
44			99.4
45			99.5
46			99.6
47			99.7
48			99.9
49			99.9
50			100.0
51			100.0
52			
53			
54			
55			
56			

*CI* Cognitive irritation, *EI* emotional irritation, *GI* general irritation.

Table 5 presents the scale means and standard deviations for different age groups and both sexes.

**Table 5:** Means and standard deviations for CI, EI and GI for different age groups, divided by sex.

Age	Sex	*n*	CI		EI		GI	
			*M*	*SD*	*M*	*SD*	*M*	*SD*
≤ 29 years	Female	119	7.44	4.53	10.89	5.81	18.32	9.51
	Male	133	6.27	3.88	9.88	5.21	16.15	8.30
	Total	252	6.82	4.24	10.36	5.51	17.18	8.94
30–39 years	Female	150	7.06	3.97	9.97	5.74	17.03	8.99
	Male	177	6.96	3.97	10.28	5.25	17.24	8.16
	Total	327	7.01	3.96	10.14	5.47	17.14	8.54
40–49 years	Female	182	6.90	4.10	10.63	6.04	17.53	9.54
	Male	175	7.04	3.95	10.60	5.62	17.65	9.05
	Total	357	6.97	4.03	10.62	5.83	17.59	9.29
50–59 years	Female	204	6.85	3.55	10.42	5.22	17.27	7.99
	Male	212	7.34	4.48	10.28	5.79	17.62	9.60
	Total	416	7.10	4.06	10.35	5.51	17.45	8.84
≥ 60 years	Female	53	6.11	3.85	9.24	5.61	15.35	9.06
	Male	75	6.94	4.07	10.87	5.86	17.80	9.17
	Total	128	6.59	3.99	10.19	5.79	16.79	9.17
All	Female	708	6.95	3.99	10.37	5.68	17.32	8.96
	Male	772	6.96	4.11	10.34	5.53	17.30	8.89
	Total	1480	6.96	4.05	10.36	5.60	17.31	8.92

*CI* Cognitive irritation, *EI* emotional irritation, *GI* general irritation.

### Comparison to the existing norms

In Mohr, Müller et al.’s sample^3^, the mean GI score was 24.8 (*sd *= 9.7) for the total sample. In the sample presented here, the overall mean is 17.3 (*sd *= 8.9). An inferential test shows a significant difference of 7.5 (*sd *= 0.29), 95% CI [6.94; 8.07]: T(5,508) = 26, *p < *0.001. In Mohr et al.'s sample, 1% of participants had an overall score of eight, which represents the lowest possible score on the IS, compared to 17.1% in the present data set.

### IS and socio-demographic data

Higher education (university degree vs. other: *T*(1,475) = 5.50, *p *=  < 0.001, *d *= 0.424; 95% CI [0.271; 0.573] for CI), former unemployment (yes vs. no: *T*(1,473) = 4.56, *p < *0.001, *d *= 0.239; 95% CI [0.135; 0.341] for EI) and higher household income (up to 2499 € vs. over 3500 €) *T*(1,055) = − 2.91, *p *= 0.004, *d *= − 0.186; 95% CI[− 0.311; − 0.061] for CI) were significantly associated with higher irritation scores (for more detailed results see Table 6), although most effect sizes were small (cohen's *d ~ *0.2). No differences were found with regard to sex and household size (living alone or with others).

**Table 6:** Group-wise comparison (Two sample t-tests).

			CI				EI				GI			
		*n*	*M*	*SD*	*p*	*d*	*M*	*SD*	*p*	*d*	*M*	*SD*	*p*	*d*
Sex	Female	708	6.95	3.99			10.37	5.68			17.32	8.96		
	Male	772	6.96	4.11	0.961	0.002	10.34	5.53	0.931	0.005	17.30	8.89	0.974	0.002
Education	University	196	8.43	4.62			10.73	5.51			19.15	9.21		
	Other	1.281	6.73	3.91	< 0.001	0.424	10.29	5.62	0.313	0.078	17.03	8.86	0.002	0.238
Unemployment	Ever	662	7.16	4.01			11.09	5.86			18.24	9.10		
	Never	813	6.80	4.09	0.094	0.089	9.76	5.30	< 0.001	0.239	16.56	8.72	< 0.001	0.189
Living alone	Yes	580	6.93	4.24			10.32	5.53			17.24	9.06		
	No	900	6.96	3.93	0.822	− 0.007	10.38	5.65	0.831	− 0.011	17.36	8.84	0.812	− 0.013
Income	< 2499	667	6.67	4.00			10.38	5.70			17.06	9.14		
	> 3500	390	7.43	4.18	0.004	− 0.186	10.35	5.51	0.921	0.005	17.78	8.79	0.210	− 0.080

*CI* Cognitive irritation, *EI* emotional irritation, *GI* general irritation, Income = household income in €. Different *n* are due to missing values.

*d* = Cohen’s d effect size.

### Convergent and discriminant validity

Table 7 shows the correlations of the IS and its subscales with clinical indicators, like the PHQ-4, different somatic complaints and psychological functioning. All correlations are significant at *p < *0.001 and show the expected direction with correlation coefficients ranging from 0.16 to 0.49. The *z*-values according to Dunn and Clark indicate whether the correlations between CI and EI with the respective scale differ significantly from each other^42^. For the PHQ-4 and the Mini-ICF-APP-S the correlations with the subscale EI are significantly higher than those with the subscale CI. For the somatic scales the calculated effect sizes indicate very small or no differences between EI and CI.

**Table 7:** Pearson correlations with 95% confidence intervalls of CI, EI and GI with depression and anxiety, psychosomatic complaints and psychological functioning.

Scale	Subscale	CI	EI	GI	CI vs. EI		
					*z*	*p*	*r*
PHQ-4	Total	0.35 (0.31;0.40)	0.46 (0.42;0.50)	0.45 (0.41;0.49)	5.92	**< 0.00**1	.15
	PHQ-2	0.32 (0.27;0.36)	0.41 (0.37;0.45)	0.40 (0.36;0.44)	5.07	**< 0.00**1	.13
	GAD-2	0.34 (0.29;0.38)	0.43 (0.39;0.47)	0.43 (0.38;0.47)	5.29	**< 0.001**	.14
BDS 25	Total	0.40 (0.35;0.44)	0.45 (0.40;0.49)	0.46 (0.42;0.50)	2.85	**0.004**	.07
	Cardiopulmonary	0.28 (0.23;0.33)	0.32 (0.28;0.37)	0.33 (0.28;0.38)	2.11	**0.035**	.05
	Gastrointestinal	0.30 (0.25;0.34)	0.32 (0.27;0.36)	0.33 (0.29;0.38)	1.11	0.268	.03
	Muscoskeletal	0.29 (0.24;0.34)	0.35 (0.30;0.39)	0.35 (0.30;0.39)	2.97	**0.003**	.08
	General	0.41 (0.36;0.45)	0.45 (0.41;0.49)	0.47 (0.43;0.51)	2.52	**0.012**	.07
SSS-8	Total	0.42 (0.38;0.46)	0.46 (0.42;0.50)	0.48 (0.44;0.52)	2.32	**0.020**	.06
	Gastrointestinal	0.26 (0.21;0.30)	0.27 (0.22;0.32)	0.29 (0.24;0.33)	0.67	0.500	.02
	Pain	0.34 (0.29;0.38)	0.39 (0.34;0.43)	0.40 (0.35;0.44)	2.60	**0.009**	.07
	Cardiopulmonary	0.26 (0.21;0.31)	0.28 (0.24;0.33)	0.30 (0.25;0.34)	1.15	0.252	.03
	Fatigue	0.42 (0.38;0.46)	0.45 (0.41;0.49)	0.48 (0.44;0.51)	1.75	0.080	.05
GSC-8	Total	0.39 (0.34;0.43)	0.49 (0.45;0.53)	0.48 (0.44;0.52)	5.71	**< 0.001**	.15
	Fatigue	0.38 (0.34;0.42)	0.48 (0.44;0.52)	0.47 (0.43;0.51)	5.40	**< 0.001**	.14
	Gastrointestinal	0.28 (0.23;0.33)	0.36 (0.31;0.40)	0.35 (0.31;0.40)	4.19	**< 0.001**	.11
	Muscoskeletal	0.32 (0.27;0.36)	0.37 (0.33;0.42)	0.38 (0.33;0.42)	3.11	**0.002**	.08
	Cardiopulmonary	0.24 (0.20;0.29)	0.36 (0.31;0.40)	0.34 (0.29;0.38)	6.12	**< 0.001**	.16
Comorbidity		0.16 (0.11;0.21)	0.16 (0.11;0.21)	0.17 (0.12;0.22)	0.05	0.960	.00
Mini-ICF-APP-S	Total	0.18 (0.13;0.23)	0.34 (0.30;0.40)	0.30 (0.25;0.34)	8.30	**< 0.001**	.21
	Cognitive and soft skills	0.13 (0.08;0.18)	0.31 (0.26;0.35)	0.25 (0.20;0.30)	9.01	**< 0.001**	.23
	Interactional skills	0.21 (0.16;0.25)	0.33 (0.28;0.37)	0.30 (0.25;0.35)	6.81	**< 0.001**	.17
	Basic skills	0.13 (0.08;0.18)	0.25 (0.20;0.30)	0.22 (0.17;0.27)	5.80	**< 0.001**	.15

*CI* Cognitive irritation, *EI* emotional irritation, *GI* general irritation, *PHQ* Patient Health Questionnaire, *GAD* General anxiety disorder, *BDS 25* Bodily Distress Syndrom Checklist 25, *SSS-8* Somatic Symptom Scale 8, *GSC-8* Giessen Subjective Complaints-8, *Mini-ICF-APP-S* International Classification of Functioning, Disability and Health rating for psychological activity and participation.

All correlations are significant on a *p*-value < 0.001.

z-Value of Dunn & Clark (1969).

*p* values show the significance of the *z*-value comparing correlations of scales with EI versus CI.

*r* = effect size of *z*;* r* = *z*/sqrt(*z*^2 + n).

Significant values are in bold.

Table 8 shows the correlations with work related stressors and with individual ressources (self-efficacy). All correlations are significant at *p < *0.001 and show the expected direction with correlation coefficients ranging from − 0.37 to 0.76. Z-Values according to Dunn and Clark^42^ show higher correlations of the scales with EI in comparison with CI for bullying and self-efficacy, although the effect sizes are small, and higher correlations of CI with OC.

**Table 8:** Pearson correlations with 95% confidence intervalls of CI, EI and GI with work related stressors and individual ressources.

Scale		CI	EI	GI	CI vs. EI		
					*z*	*p*	*r*
MOB-C		0.26 (0.21;0.30)	0.33 (0.29;0.38)	0.32 (0.28;0.37)	3.94	**< 0.001**	.10
OC		0.76 (0.75;0.80)	0.59 (0.55;0.62)	0.72 (0.69;0.74)	14.34	**< 0.001**	.35
GSE-EL		− 0.31 (− 0.35;− 0.26)	− 0.37 (− 0.41;− 0.33)	− 0.37 (− 0.41;− 0.33)	3.42	**< 0.001**	.09

*CI* Cognitive irritation, *EI* emotional irritation, *GI* general irritation, *MOB-C* mobbing intensity of colleagues, *OC* overcommitment, *GSE-EL* general self-efficacy in easy language.

All correlations are significant on a *p*-value *p* < 0.001.

*z*-Value of Dunn and Clark (1969).

*p* of *z*-value values show the significance of the *z*-value comparing correlations of scales with EI versus CI.

*r* = effect size of *z*; *r* = *z*/sqrt(z^2^ + n).

Significant values are in bold.

## Discussion

Our results suggest that the previous normative data of the IS^6^ may have overestimated the manifestations of irritation in the labour force. Compared with the previous data collected in 2005, the new normative data show a more positively skewed distribution with lower means. The sample used by Mohr, Müller et al. contains a large proportion (26.33%) of firefighters and police officers—a group with high job strain^43–45^. Moreover, one study which accounted for 17% of the sample was conducted in Eastern Germany at the time of German reunification, a period that brought many stressors and an increased risk of unemployment for residents of East Germany^17^. Thus, the lower means found in this representative sample presented here are plausible, and should provide a more accurate information on the level of irritation in the German labour force.

From a practical point of view, the higher mean values presented 2005 thus contributed to an underestimation of the average level of irritation in organizations. This deficiency has been corrected with the present new normative data. Our study allows for the first time reliable statements on the relationship between IS and important socio-demographic variables such as education, income, age and gender: The correlations with demographic characteristics indicate that higher education and higher income are related with higher levels of irritation, in particular with cognitive irritation. While high social status is often associated with better mental health^46^, it is also associated with more job responsibilities and stress^47^, which may explain the association with cognitive irritability.

In contrast to Mohr, Müller et al., we found no age differences with respect to CI. It is possible that the relationship between age and CI is moderated by type of employment and gender, as found by Rauschenbach et al.^7^, and in specific populations^7^.

Unemployment is generally associated with reduced health, including mental illness^48–50^. Accordingly, individuals in this sample who were previously unemployed show higher EI scores than individuals who were never unemployed. Even when employment continues again, the experience of unemployment seems to negatively affect health^51^.

Our findings largely confirm the assumption derived from the theory of goal discrepancy^15^ that EI represents a more severe impairment of well-being compared to CI. EI seems to capture more significant distress compared to CI because it captures the emotional strain and not only the mental preoccupation with work, which explains the stronger correlations with the other mental impairment scales (e.g. PHQ-4, Mini-ICF-APP-S, etc.). Perceived anger, nervousness and irritability (components of EI) are experienced rather physically, which could explain the higher correlations with the somatic scales with the EI compared to the CI. Causality cannot be assumed, but rather an interdependence of the components^49,52^.

The correlations with the assessed health indicators confirm overall the construct validity of the Irritation Scale. The GI as well as the subdimensions CI and EI correlate consistently with other indicators of mental well-being, functioning, and physical and mental health in the expected direction. Moreover, the experience of bullying as a main work stressor^53,54^ is also significantly positively related with higher levels of irritation.

We reviewed the factor structure of the IS scale using the available data. The findings of the factor analysis point to a two-factorial structure of the scale, although the fit of the two-factorial model in the CFA is slightly poorer, than that calculated by Müller et al.^16^, and shows contradictory fit. The two-factorial model should not be rejected because of its indices. Many authors prefer the use of fit indices for a differentiated consideration of possible model strengths and weaknesses^55^, or, in particular, for a comparison of models^56^, rather than simply applying strict cut-offs^57,58^. According to RMSEA the two-factorial model reasonably match the *empirical data,* though the CFI indicates an insufficient fit of the hypothesized model compared to a *null model*, i.e. a potential model with zero fit. A bifactorial model that would represent the theoretical assumptions of the irritation construct with one general factor and two underlying subfactors EI and CI did not converge, due to negative error covariances. Eid et al.^59^ reports that bifactorial models frequently result in improper solutions and misspecifications, because main assumptions of bifactorial models are often not met, i.e. uncorrelated first order factors, loadings of observed variables on first-order factors as well as the general factor. Post-hoc analyses indicate that the bifactorial model in our study can be specified if a loading on the GI is not allowed for some items. Improving the operationalization of the GI should therefore be the subject of further research.

Overall, the presented strong factor loadings and the plausible correlation patterns of the EI and CI subfactors with other constructs support the reasonable interpretation of the two subscales, although the CFA results suggest that the factor structure of the irritation scale requires further investigation. Unfortunately, we could not finally clarify with the performed CFA how the additional overall factor GI can be exactly operationalized. Therefore, its interpretation should be taken with caution.

### Limitations

This is a cross-sectional study. No causality can be derived from the correlations with the other scales presented here. Moreover, apart from bullying, no further information on the job and working conditions was collected so that no further correlations with irritation can be examined.

## Conclusion

The Irritation Scale (IS) is recommended for the asessment of work-related mental strain and is particularly valuable for the early detection of a potential deterioration of (mental) health. The present study corroborated the construct validity of the IS. With the new normative data presented here, an even more accurate classification of IS values in respect to the general German working population is possible. Underestimation of the average level of irritation in organisations can be avoided in the future.

### Supplementary Information

Supplementary Table 1.

## Data Availability

Preregistration of Studies and Analysis Plans: This study was not preregistered. The datasets used and/or analyzed during the current study are available from the following authors on reasonable request:: IS and OC: HG; GSE-EL: BS; MOB-C: JK; BDS 25 and SSS-8 and Comorbidity Scale: WH; GSC-8 and PHQ-4: EB; Mini-ICF-APP (in aggregate form): BM.
