# Supplementary material for: Validation of the irritation scale on a representative German sample: new normative data
Source: Sci Rep. 2023 Sep 16;13:15374. doi: 10.1038/s41598-023-41829-4 (PMC10505143; doi:10.1038/s41598-023-41829-4)
Supplement: Supplementary file 1 — Supplementary Table 1. [file 41598_2023_41829_MOESM1_ESM.docx]

**Validation of the Irritation Scale on a representative German sample:**

**New normative data**

**Electronic Supplementary Material**

**Supplementary table**

*Sample characteristics including sex, age, education, marital status, employment and income (N = 2,531)*

|  | *N* | % |
| --- | --- | --- |
| Biological sex Female | 1,350 | 53.3 |
| Male | 1,181 | 46.7 |
| Age (*Mean* = 48.44, *sd* = 17.86) |  |  |
| 14-19 | 141 | 5.6 |
| 20-29 | 319 | 12.6 |
| 30-39 | 386 | 15.3 |
| 40-49 | 416 | 16.4 |
| 50-59 | 511 | 20.2 |
| 60-69 | 432 | 17.1 |
| 70-79 | 241 | 9.5 |
| 80-95 | 85 | 3.4 |
| Education level  No graduation or student | 135 | 5.33 |
| Lower secondary school certificate | 689 | 27.2 |
| Secondary school certificate | 1,123 | 44.4 |
| Upper secondary school certificate | 323 | 12.8 |
| University degree | 251 | 9.9 |
| Other degree or no specification | 10 | 0.4 |
| Marital status  Married and living together | 1,101 | 43.5 |
| Married and living apart | 70 | 2.8 |
| Single | 760 | 30 |
| Divorced | 368 | 14.5 |
| Widowed | 220 | 8.7 |
| No specification | 12 | 0.5 |
| Employment  Full-time work (35 hours and more) | 1,141 | 45.1 |
| Part-time work (15-34 hours) | 287 | 11.3 |
| Work less than 15 hours | 73 | 2.9 |
| Pensioner, retiree, early retirement, in short-time work, federal voluntary service,  maternity leave/parental leave or (currently) not employed | 810 | 32.0 |
| Vocational training or no specification | 220 | 8.7 |
| Monthly household income in € |  |  |
| < 1500 | 626 | 24.7 |
| 1500-2499 | 783 | 30.9 |
| 2500-3499 | 552 | 21.8 |
| > 3500 | 513 | 20.3 |
| No specification | 57 | 2.2 |
